# Dietary quality of school meals and packed lunches: a national study of primary and secondary schoolchildren in the UK

**DOI:** 10.1017/S1368980022001355

**Published:** 2022-06-01

**Authors:** Erin Haney, Jennie C Parnham, Kiara Chang, Anthony A Laverty, Stephanie von Hinke, Jonathan Pearson-Stuttard, Martin White, Christopher Millett, Eszter P Vamos

**Affiliations:** 1 Public Health Policy Evaluation Unit, School of Public Health, Imperial College London, London W6 8RP, UK; 2 School of Economics, University of Bristol, Bristol, UK; 3 Erasmus School of Economics, Erasmus University Rotterdam, Rotterdam, The Netherlands; 4 Department of Epidemiology and Biostatistics, School of Public Health, Imperial College London, London, UK; 5 Northumbria Healthcare NHS Foundation Trust, Newcastle upon-Tyne, UK; 6 Health Analytics, Lane Clark & Peacock LLP, London, UK; 7 MRC Epidemiology Unit, University of Cambridge, Cambridge, UK; 8 Public Health Research Centre & Comprehensive Health Research Center (CHRC), National School of Public Health, Lisbon, Portugal

**Keywords:** School meals, Child nutrition, Packed lunches, Adolescent nutrition

## Abstract

**Objective:**

School lunches represent a key opportunity to improve diets and health of schoolchildren. No recent nationally representative studies have examined the nutritional differences between school meals and packed lunches in the UK. This study aimed to characterise and compare the nutritional quality of school meals and packed lunches among primary and secondary school-age children.

**Design:**

A pooled cross-sectional analysis of the UK’s National Diet and Nutrition Survey (2008–2017).

**Setting:**

United Kingdom.

**Participants:**

3001 children (aged 4–16 years) who completed a 3/4-d food diary which recorded meal type (school meal/packed lunch). Multivariable logistic regression models assessed associations of meeting food and nutrient recommendations by meal type. Analyses were stratified by academic key stages (KS).

**Results:**

KS-1 (4–7 years) and 2 (8–11 years) children consuming school meals were more likely to meet minimum recommendations for vegetables, protein-rich foods and fibre, and not exceed maximum recommendations for salt, savoury and sweet snacks compared with pupils consuming packed lunches. However, in KS-3 (12–14 years) and 4 (14–16 years), these effects were reduced. As children aged, the median weight of fruits, vegetables, protein-rich foods and dairy products consumed typically decreased for both school meals and packed lunches, and generally an increasing proportion of school meals contained sweet and savoury snacks.

**Conclusion:**

These findings suggest school meals are nutritionally superior to packed lunches but are not yet optimal. Quality declined at higher KS. Actions to improve lunches of primary and secondary schoolchildren across the UK are needed, with attention to KS-3 and 4 in secondary schools.

Maintaining a healthy and balanced diet is essential for the prevention of childhood obesity and its long-term consequences^(1)^. However, suboptimal dietary patterns have been widely documented among children in the UK^(2,3)^. Less than 15 % of school-age children meet the UK government’s five-a-day target for fruit and vegetables^(2)^, the average daily intake of sugar is over double the recommended level^(2)^ and mean fibre intake is below recommended levels in all age groups^(4)^. Longitudinal studies indicate that dietary quality declines as children enter adolescence^(5,6)^, with the consumption of sugary drinks increasing and the consumption of fruit and vegetables reducing^(5,6)^. As both diet and obesity have been shown to track from childhood to adulthood^(7)^, adopting healthy eating behaviours from childhood is critical to positively influence health across the lifespan.

A survey conducted in 2013–2014 showed that 42·5 % of schoolchildren in the UK regularly consume a lunch provided by their school^(8)^. Schools represent a critical element of a child’s food environment as they consume one-third of their weekday food at school^(9)^. As such, school lunches provide an important opportunity for improving children’s diet quality and eating behaviours in the school environment that may also contribute to the quality of their overall diet^(9,10)^. School Food Standards first became part of the public health agenda across all four countries in the UK in 2001 and were revised and updated between 2007 and 2015^(10,11)^. By September 2013, compulsory School Food Standards were in place for primary and secondary schools (except for academies in England) across all countries in the UK to ensure food provided by schools is of high quality, healthy and nutritious and protects vulnerable individuals, such as children from low-income families^(10)^. The guidelines consider the provision of foods and beverages and provide recommended types and portion sizes of fruits and vegetables, starchy foods, protein-rich foods, dairy products and restrictions on foods high in sugar, salt and fat^(11–14)^. Previous research indicated that the introduction of the School Food Standards in England has overall increased the dietary quality of school meals^(15,16)^. Furthermore, a meta-analysis of similar school-based regulations worldwide reported they were associated with improved dietary quality^(17)^. Analyses of 1990–2011 data have shown that school meals are of higher nutritional quality than packed lunches among primary schoolchildren^(9,18–20)^, with only a small proportion of packed lunches meeting the School Food Standards^(21)^. Similar results were observed in secondary schools; however, a smaller difference was typically observed between school meals and packed lunches^(22–25)^.

Despite existing evidence comparing the nutritional gap between school meals and packed lunches among primary^(9,18–20)^ and secondary school students^(22–25)^, there have been no studies analysing data collected within the past decade, despite the revision and updating of nutritional guidelines during this period^(11)^. Additionally, previous studies were not UK wide and did not compare primary and secondary schoolchildren. Furthermore, addressing this gap in research is crucial since all schoolchildren aged 4–7 years in England and Scotland are now offered universally free school meals, a programme which is associated with increased school meal uptake^(26)^ and has recently been expanded in Scotland to include children aged up to 9 years^(27)^. Therefore, this study aimed to use detailed nationally representative dietary survey data from the UK (2008–2017) to compare the diet and nutritional quality of school meals and packed lunches.

## Methods

This study used pooled cross-sectional data obtained from the National Diet and Nutrition Survey (NDNS) rolling programme, collected between 2008 and 2017. The NDNS is an annual survey used to assess the diet of the UK population aged 1·5 years and over^(2)^. The survey draws a stratified random sample from the national postcode registry to produce a nationally representative sample of individuals within households covering England, Wales, Scotland and Northern Ireland^(2)^. Data were publicly accessible and obtained from the UK Data Service. All NDNS participants aged 4–16 years attending primary or secondary school were included (*n* 4677). Survey years 10–11 (2018–2019) were not available at the time of analysis.

### Diet and nutritional outcomes

Diet was measured in the NDNS using a food diary of four consecutive days, including at least one weekend day^(2)^, to capture the totality of the diet. Diaries were initially validated by the NDNS team against repeated 24-h recalls in a subset of participants aged 4 years and older. Dietary intake collected by food diaries was found to be comparable to 24-h recalls, but the food diary was considered more flexible and appropriate for use in young children^(28)^. Diaries were self-completed for children 12 years and over and by a carer for children less than 12 years. Age-appropriate photographs of frequently consumed foods were used to assist participants in quantifying food intake alongside weights from food labels^(28)^. Data on food and drink consumed were linked to the Department of Health’s Nutrient Databank to derive their nutrient content. In this secondary analysis of the NDNS data, only food and drink variables consumed for a school lunch were included in the outcome variables. Food items were defined as a school lunch if they occurred on a Monday to Friday between 12:00 and 14:00 on school premises, therefore morning, evening and all weekend items were excluded. Participants were excluded if they did not record a lunch (*n* 1558). All items consumed as part of a school lunch were summed and averaged per school day by participant. The total number of school lunches recorded by participants varied from 1 to 3 d (1 d (*n* 457), 2 d (*n* 1289) and 3 d (*n* 1255)).

Seven food groups (fruits, vegetables, protein-rich foods (meat, fish, eggs and beans), wholemeal products (wholemeal bread, brown rice), dairy products (milk, yogurt and cheese), savoury snacks and sweet snacks) were chosen to reflect categories referred to in the 2014 English School Food Standards (definitions are presented in online supplementary material, Appendix A). Seven nutrient variables (fibre (g), vitamin C (mg), Ca (mg), Fe (mg), non-milk extrinsic sugar (NMES (g) and salt (g)) reflected the nutrient guidance in the 2009 Standards^(29)^. Nutrient-based guidelines were phased out by the Government in 2015 in favour of food-based standard which were more practical to implement. However, the nutrient-based guidelines are used in this study as they provide a useful benchmark for optimal lunchtime nutritional intake for children. Nutrient variables were dichotomised into whether they met the age-specific minimum or maximum nutrient standards (thresholds are detailed in online supplementary material, Appendix B). Intakes of food groups were assessed as both continuous variables (g/lunch) averaged across school days and dichotomised variables indicating no (0 g/lunch) or some (>0 g/lunch) intake.

### Meal type

The diet diaries indicated where an item was consumed and whether it was ‘food from home’, which was categorised as a ‘packed lunch’, or ‘bought at the canteen’ (includes free school meals), which was categorised as a ‘school meal’. If the meal type of a school lunch was not recorded (*n* 325), the survey question ‘on a school/college day, what do you/does (child’s name) usually have for lunch?’ was used to determine the child’s meal type category. Participants were classified as school meal consumers if they responded, ‘cooked school meal’ or ‘cold school meal (including sandwiches, salads)’ and were coded as packed lunch consumers if they replied, ‘packed lunch (from home)’. Participants who could not be clearly defined as bringing food from home or from school were excluded (*n* 116).

### Study covariates

Covariates included were survey year (2008–2017), sex (male/female), age (years), ethnicity (White/non-White), equivalised household income (tertiles), a measure of household income that takes account of the differences in a household’s composition and country (England, Scotland, Northern Ireland or Wales). Survey year effects will capture any variation due to the Universal Infant Free School Meal (UIFSM) policy introduced in 2014 in England and in 2015 in Scotland that offers free school meals for all children aged 4–7 years in state-funded primary schools. Participants with missing ethnicity were excluded (*n* 2). Equivalised household income was imputed for participants with missing data (*n* 137) using ten iterations of the classification and regression trees (CART) method^(30)^ in R.

### Statistical analysis

Bivariate significance tests were used to assess sample characteristics across meal types (school meal *v*. packed lunch), separately for each academic key stage (key stage 1 (KS-1), aged 4–7 years; key stage 2 (KS-2), 8–11 years; key stage 3 (KS-3), 12–14 years; key stage 4 (KS-4), 15–16 years). For children of each KS, we compared diet and nutritional outcomes between meal types consumed, using the non-parametric Wilcoxon–Mann Whitney tests for continuous outcomes and *χ* ^2^ tests for dichotomised outcomes, as appropriate.

Multivariable logistic regression models were used to evaluate the association between meal type (school meals = 0, packed lunch = 1) and the likelihood of students consuming each food group (fruits, vegetables, protein-rich foods, wholemeal products, dairy products, savoury snacks and sweet snacks) and meeting nutrient-based standards (fibre, vitamin C, Ca, Fe, NMES and salt). Models were adjusted for all covariates. The analyses were then stratified by KS.

Sensitivity analyses were performed to check for robustness. First, we excluded any participants whose meal type was based on the survey question, ‘on a school/college day, what do you/does (child’s name) usually have for lunch?’ to test whether the results were robust against potential misclassification bias. Second, we assessed the potential dietary misreporting bias using the Goldberg method, adapted for children^(31,32)^. Participants’ estimated energy requirements were calculated (using Schofield equations) and compared with their reported energy intake using the Goldberg cut-offs, and we excluded 485 (15 %) who may have over- or under-reported their dietary intake.

All statistical analyses were performed using Stata, version 15.0, unless stated otherwise. Survey weights provided by the NDNS were applied in all data analyses to account for sampling and non-response bias^(2)^. *P*-values of <0·05 were considered statistically significant for all tests.

## Results

A total of 3001 children were included in the analysis (Table 1). A similar proportion of students consumed a school meal *v*. a packed lunch in KS 1–4, for example, 51 % of KS-1 students and 49 % of KS-4 students consumed a school meal. Gender and ethnicity patterns by meal types were broadly similar across KS. However, children in the lowest income tertile were more likely to consume a school meal than a packed lunch regardless of age.

**Table 1:** Unweighted sample characteristics of participants included in the study

Characteristic	Units	Primary school								Secondary school								Total	
		Key stage 1				Key stage 2				Key stage 3				Key stage 4					
		School meal		Packed lunch		School meal		Packed lunch		School meal		Packed lunch		School meal		Packed lunch			
Participants	*n* (%)	498	52·5	450	47·5*	469	45·9	553	54·1	364	52·6	328	47·4	165	48·7	174	51·3*	3001	100
Age (years)	Mean (sd)	5·7	1·0	5·7	1·0	9·5	1·1	9·5	1·1	13·0	0·8	13·0	0·8	15·4	0·5	15·5	0·5	9·8	3·5
Sex	*n* (%)																		
Male		251	50·4	247	54·9	254	54·2	287	51·9	186	51·1	163	49·7	76	46·1	68	39·1	1532	51·1
Ethnicity	*n* (%)							*											
White		428	85·9	392	87·1	394	84·0	500	90·4	324	89·0	295	89·9	139	84·2	153	87·9	2623	87·5
Survey year	*n* (%)			*															
1 (2008–2009)		58	11·7	65	14·4	69	14·7	75	13·6	54	14·8	30	9·2	16	9·7	20	11·5	387	12·9
2 (2009–2010)		49	9·8	57	12·7	46	9·8	75	13·6	43	11·8	51	15·6	20	12·1	23	13·2	364	12·1
3 (2010–2011)		43	8·6	49	10·9	53	11·3	71	12·8	44	12·1	36	11·0	25	15·2	28	16·1	349	11·6
4 (2011–2012)		56	11·2	66	14·7	64	13·7	77	13·9	48	13·2	52	15·9	26	15·8	17	9·8	406	13·5
5 (2012–2013)		29	5·8	55	12·2	39	8·3	33	6·0	28	7·7	29	8·8	15	9·1	24	13·8	252	8·4
6 (2013–2014)		54	10·8	62	13·8	44	9·4	56	10·1	48	13·2	43	13·1	21	12·7	22	12·6	350	11·7
7 (2014–2015)		55	11·0	39	8·7	48	10·2	45	8·1	30	8·2	29	8·8	12	7·3	13	7·5	271	9·0
8 (2015–2016)		74	14·9	24	5·3	51	10·9	67	12·1	37	10·2	23	7·0	17	10·3	19	10·9	312	10·4
9 (2016–2017)		80	16·1	33	7·3	55	11·7	54	9·8	32	8·8	35	10·7	13	7·9	8	4·6	310	10·3
Country	*n* (%)			*								*							
England		317	63·7	246	54·7	277	59·1	324	58·6	196	53·9	217	66·2	86	52·1	111	63·8	1774	59·1
Northern Ireland		57	11·5	62	13·8	63	13·4	81	14·7	67	18·4	49	14·9	31	18·8	23	13·2	433	14·4
Scotland		59	11·9	72	16·0	71	15·1	88	15·9	52	14·3	29	8·8	21	12·7	16	9·2	408	
Wales		65	13·1	70	15·6	58	12·4	60	10·9	49	13·5	33	10·1	27	16·4	24	13·8	386	
Equivalised household income	*n* (%)			*				*								*			
1^st^ tertile (lowest)		162	32·5	127	28·2	199	42·4	153	27·7	135	37·1	112	34·2	66	40·0	50	28·7	1004	33·5
2^nd^ tertile		150	30·1	172	38·2	137	29·2	207	37·4	105	28·9	114	34·8	57	34·6	60	34·5	1002	33·4
3^rd^ tertile (highest)		186	37·4	151	33·6	133	28·4	193	34·9	124	34·1	102	31·1	42	25·5	64	36·8	995	33·2

BAME, black, Asian, minority, ethnic; IMD, index of multiple deprivation.

* Significant *P*-value <0·05 for difference between school meal and packed lunch. χ^2^ statistic used for testing differences in proportions and t-test statistic used for test differences in mean across meal types.

Overall, across KS (percentages indicate range across KS 1–4), compared with packed lunches, a larger proportion of school meals contained vegetables (71·5–92·4 % *v*. 39·5–46·0 %) and protein-rich foods (77·8–91·3 % *v*. 68·4–79·4 %) while a smaller proportion contained fruit (30·2–70·2 % *v*. 55·2–86·9 %), wholemeal products (8·1–13·7 % *v*. 30·8–41·3 %), savoury snacks (3·3–11·2 % *v*. 40·0–45·4 %) and sweets snacks (30·2–45·1 % *v*. 58·1–76·2 %) (Table 2). These findings were consistent across age groups except for dairy products, when compared with packed lunches, a smaller proportion of KS-1 (63·1 % *v*. 78·2 %) and KS-2 (65·2 % *v*. 73·8 %) school meals contained dairy products, whereas there was no significant difference in the proportion in KS-3 and KS-4. The median amount eaten of each of these food groups followed the same pattern with school meals containing larger portions of vegetables and protein-rich foods, and smaller portions of fruit, wholemeal products, savoury snacks and sweet snacks (Table 2). At higher KS, however, the proportion of meals containing fruits, vegetables, protein-rich foods, and dairy products and the median amount consumed generally decreased for both school meals and packed lunches. For instance, the median portion of fruit and vegetables eaten in school meals decreased from 6·8 g and 34·5 g in KS-1 to 0 g and 14·0 g in KS-4, respectively. Additionally, as children aged, a greater proportion of school meals contained sweet and savoury snacks. In KS-1, 3·3 % of school meals contained savoury snacks, compared with 11·2 % in KS-4. The proportions were relatively consistent in packed lunches across KS with the proportion consuming sweet and savoury snacks varying between 40·0–45·4 % and 58·1–76·2 %, respectively.

**Table 2:** Children consuming each food group (%) and median weight (g) and interquartile range of each food group consumed in school meals for 1496 pupils and packed lunches for 1505 pupils, results adjusted using survey weights (*n* 3001)

Food type	Key stage 1								Key stage 2							
	Amount eaten (g)					Proportion eating			Amount eaten (g)					Proportion eating		
	School meal		Packed lunch		*P* *	School meal	Packed lunch	*P* †	School meal		Packed lunch		*P* *	School meal	Packed lunch	*P* †
	Median	IQR	Median	IQR		%	%		Median	IQR	Median	IQR		%	%	
Fruit (g)	6·8	0, 29·6	34·5	7·31, 68·77	<0·001	70·2	86·9	<0·001	2·3	0, 30·1	25·0	1·7, 70·0	<0·001	58·2	79·0	<0·001
Vegetables (g)	34·4	19·7, 54·9	0	0, 12·0	<0·001	92·4	39·5	<0·001	29·5	12·2, 52·4	0	0, 16·3	<0·001	88·5	46·0	<0·001
Protein-rich foods‡ (g)	34·2	20·7, 51·0	21·7	3·7, 33·0	<0·01	91·3	76·7	<0·001	33·5	18·5, 52·0	21·1	9·0, 34·0	<0·001	89·9	79·4	<0·001
Wholemeal products (g)	0	0, 0	0	0, 36·0	<0·001	8·1	41·3	<0·001	0	0, 0	0	0, 37·3	<0·001	9·3	37·9	<0·001
Dairy products§ (g)	6·3	0, 43·08	30·0	4·8, 54·0	<0·001	63·1	78·2	<0·001	8·65	0, 33·3	24·9	0, 55·0	<0·001	65·2	73·8	<0·001
Savoury snacks (g)	0	0, 0	0	0, 12·5	<0·001	3·3	40·0	<0·001	0	0, 0	0	0, 15·5	<0·001	7·2	44·2	<0·001
Sweet snacks (g)	0	0, 5·2	12·0	0, 30·0	<0·001	30·2	58·1	<0·001	0	0, 12·3	20·7	3·5, 55·5	<0·001	38·8	76·2	<0·001
	Key stage 3								Key stage 4							
Fruit (g)	0	0, 2·7	7·1	0, 66·7	<0·001	30·24	61·2	<0·001	0	0, 3·0	1·2	0, 47·7	<0·001	37·1	55·2	<0·001
Vegetables (g)	15·5	0, 36·6	0	0, 14·0	<0·001	71·54	44·8	<0·001	14·0	0, 34·62	0	0, 13·7	<0·001	74·4	43·0	<0·001
Protein-rich foods‡ (g)	30·0	12·2, 49·6	21·8	0, 38·5	<0·001	79·77	70·7	0·02	31·2	12·5, 45·0	20·0	0, 40·2	<0·001	77·8	68·4	0·23
Wholemeal products (g)	0	0, 0	0	0, 24·8	<0·001	13·25	32·2	<0·001	0	0, 0	0	0, 26·7	<0·001	14·7	30·8	<0·001
Dairy products§ (g)	0	0, 16·0	0	0, 21·0	0·68	48·56	48·0	0·87	3·3	0, 22·0	0	0, 21·0	0·99	52·9	46·4	0·73
Savoury snacks (g)	0	0, 0	0	0, 14·0	<0·001	9·99	45·4	<0·001	0	0, 0	0	0, 16·4	<0·001	11·2	42·4	<0·001
Sweet snacks (g)	0	0, 82·5	18·8	0, 67·2	<0·001	46·53	66·1	<0·001	0	0, 130·0	14·00	0, 80·0	0·09	45·1	60·9	0·01

* *P*-value derived from Wilcoxon–Mann Whitney assessment testing for differences between median weight eaten across meal types.

† *P*-value derived from χ^2^ statistic testing for differences in the proportion of pupils consuming each food group across meal types.

‡ Protein-rich foods include meat, fish, eggs and beans.

§ Dairy products include yogurt, milk and cheese.

Analyses of nutrient consumption did not reveal consistent patterns across KS. In the younger KS (1–2), compared with packed lunches, a significantly larger proportion of school meals met the minimum recommended intake for fibre (57·0–57·9 % *v*. 25·4–44·3 %) and did not exceed the maximum recommendation for NMES (71·7–74·1 % *v*. 47·1–68·2 %), SFA (67·5–67·6 % *v*. 49·7–75·0 %) or salt (61·9–65·9 % *v*. 30·2–61·5 %) (Table 3). Proportions of school meals and packed lunches meeting nutritional recommendations had similarly declined in KS-3 and 4. Further, there were no significant differences observed in either the proportion of students meeting the recommendations or the median amount of each nutrient consumed, except for salt in KS-3.

**Table 3:** Children meeting nutrient recommendations (%), and median weight and interquartile range consumed in school meals for 1496 pupils and packed lunches for 1505 pupils, results adjusted using survey weights (*n* 3001)

Nutrient	Key stage 1								Key stage 2							
	Amount eaten					Proportion meeting recommendation			Amount eaten					Proportion meeting recommendation		
	School meal		Packed lunch		*P* *	School meal	Packed lunch	*P* †	School meal		Packed lunch		*P* *	School meal	Packed lunch	*P* †
	Median	IQR	Median	IQR		%	%		Median	IQR	Median	IQR		%	%	
Fibre (g)	4·6	3·5, 6·05	3·9	2·8, 5·05	<0·001	57·9	44·3	<0·001	4·7	3·6, 6·0	4·0	3·0, 5·4	<0·001	57·0	41·5	<0·001
Vitamin C (mg)	14·7	8·5, 22·7	18·1	7·8, 41·1	<0·01	66·1	67·1	0·49	14·1	8·0, 25·4	18·4	6·8, 42·9	0·07	62·7	62·8	0·63
Ca (mg)	160·8	105·0, 240·2	210·0	158·0, 301·3	<0·001	38·4	61·1	<0·001	171·8	109·8, 245·8	238·1	169·1, 338·4	<0·001	31·2	55·1	<0·001
Fe (mg)	1·9	1·5, 2·4	2·0	1·6, 2·5	0·53	9·7	9·8	0·94	2·1	1·6, 2·5	2·2	1·8, 2·9	0·06	12·2	15·9	0·13
NMES (g)	9·9	4·7, 15·6	14·7	8·4, 23·2	<0·001	74·1	53·7	<0·001	10·8	4·8, 17·0	17·0	10·7, 27·4	<0·001	71·7	47·1	<0·001
SFA (g)	5·3	3·7, 7·3	6·1	4·2, 8·2	<0·001	67·6	55·5	<0·001	5·4	3·8, 7·9	6·9	4·5, 9·3	<0·001	67·5	49·7	<0·001
Salt (g)	1·1	0·8, 1·4	1·5	1·2, 2·0	<0·001	61·9	31·0	<0·001	454·2	340·1, 631·8	668·3	508·0, 859·0	<0·001	65·9	30·2	<0·001
	Key stage 3								Key stage 4							
Fibre (g)	3·9	2·7, 5·4	3·9	2·6, 5·2	0·59	29·6	25·4	0·87	4·0	3·1, 5·4	4·0	2·3, 5·8	0·92	29·8	31·5	0·32
Vitamin C (mg)	9·4	2·5, 25·9	10·5	2·3, 26·0	0·28	41·4	44·1	0·21	11·1	2·5, 2 5·0	8·39	2·6, 26·4	0·33	43·3	37·0	0·24
Ca (mg)	157·5	95·2, 247·0	177·7	109·5, 272·4	0·01	12·5	14·5	0·95	185·5	115·81, 289·74	168·0	108·4, 272·6	0·67	19·4	14·4	0·72
Fe (mg)	2·10	1·5, 2·8	2·0	1·5, 2·7	0·39	1·7	3·0	0·64	2·1	1·6, 2·7	2·2	1·3, 2·9	0·39	0·4	2·7	0·20
NMES (g)	13·6	4·0, 24·8	10·90	4·38, 22·7	0·64	63·5	68·2	0·82	12·8	1·0, 31·8	13·3	2·9, 27·3	0·83	62·1	64·1	0·73
SFA (g)	5·4	3·0, 9·0	5·13	2·77, 7·9	0·88	66·2	75·0	0·76	5·8	3·4, 10·0	5·4	2·2, 9·1	0·24	63·5	67·4	0·57
Salt (g)	540·5	350·2, 752·5	618·38	429·8, 850·8	<0·01	69·8	61·5	<0·01	1·6	1·1, 1·9	1·5	1·0, 2·2	0·25	68·0	58·3	0·06

* *P*-value derived from Wilcoxon–Mann Whitney assessment testing for differences between median weight eaten across meal types.

† *P*-value derived from χ^2^ statistic testing for differences in the proportion of pupils consuming each food group across meal types.

**Fig. 1:**
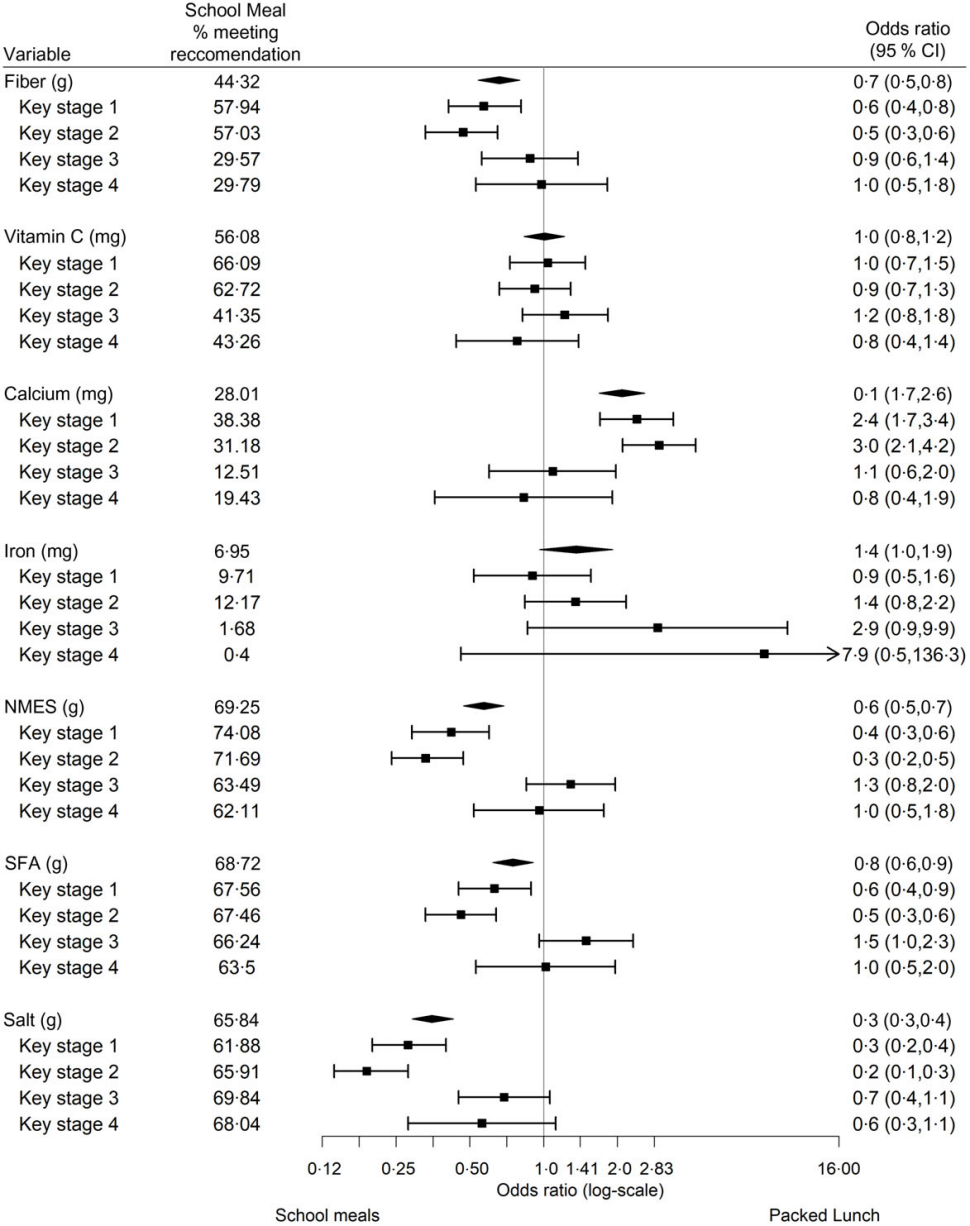
Adjusted OR (95 % CI) for the likelihood of packed lunches *v*. school meals in containing each food group, by academic key stage

Regression analyses found packed lunches were less likely to contain vegetables (adjusted OR (AOR) 0·1, 95 % CI 0·1, 0·2) and protein-rich foods (AOR 0·5, 95 % CI 0·3, 0·6) than school meals but were more likely to contain fruit (AOR 2·8, 95 % CI 2·2, 3·4), wholemeal products (AOR 4·9, 95 % CI 3·8, 6·4), dairy products (AOR 1·3, 95 % CI 1·1, 1·6), savoury snacks (AOR 10·1, 95 % CI 7·5, 13·5) and sweet snacks (AOR 3·1, 95 % CI 2·6, 3·8) (Fig. 1). When stratified by KS, effect sizes in KS-3 and 4 were smaller or non-significant compared with in KS-1 and 2. For example, in KS-1 the likelihood of packed lunches containing vegetables compared with school meals was AOR 0·05 (95 % CI 0·03, 0·08), but in KS-4 the likelihood was AOR 0·23 (95 % CI 0·13, 0·42).

**Fig. 2:**
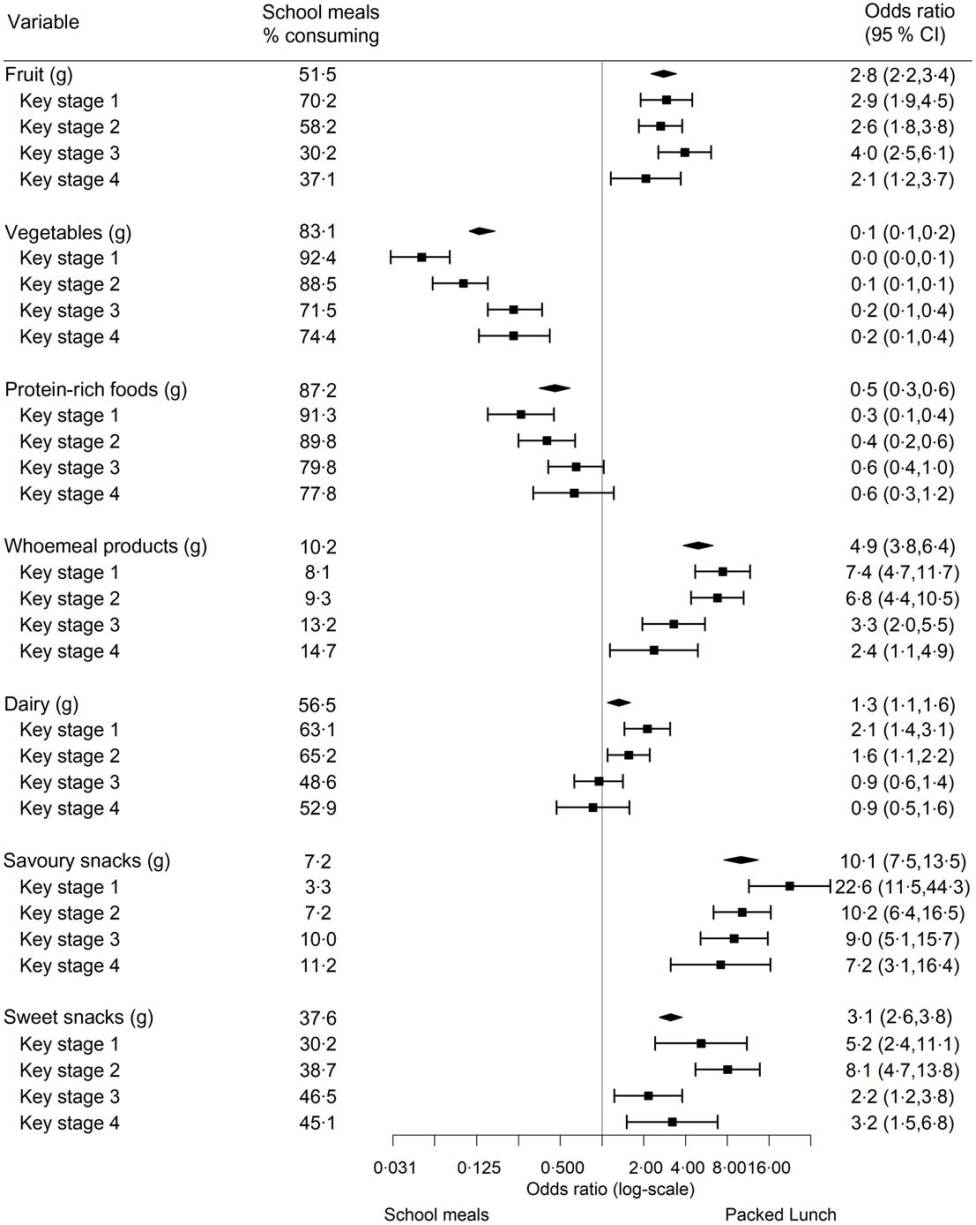
Adjusted OR (95 % CI) for the likelihood of packed lunches *v*. school meals in meeting nutrient-based outcomes, by academic key stage

Regression analyses found packed lunches were less likely than school lunches to meet nutrient intake recommendations for fibre (AOR 0·7, 95 % CI 0·5, 0·8), NMES (AOR 0·6, 95 % CI 0·5, 0·7), SFA (AOR 0·8, 95 % CI 0·6, 0·9) and salt (AOR 0·3, 95 % CI 0·3, 0·4) but were more likely to meet the Ca recommendation (AOR 2·1, 95 % CI 1·7, 2·6) (Fig. 2). When stratified by KS, significant differences between school meals and packed lunches for the likelihood of meeting nutrient intake recommendations were present in KS-1 and KS-2 for the same nutrients as the overall sample. The effect size for older children in KS-3 and 4 was smaller compared with KS-1 and 2 with no statistically significant differences present across any nutrients (Fig. 2). For example, the recommendation for fibre was significantly more likely to be met by those consuming school meals compared with packed lunches in KS-1 and 2 (AOR 0·6 (95 % CI 0·4, 0·8) and AOR 0·5 (95 % CI 0·3, 0·6), respectively). In KS-3 and 4, however, the odds of meeting the recommendation were not significantly different between meal types.

The sensitivity analysis demonstrated that results were robust to any changes in meal-type definition (online supplementary material, Appendices C and D) and after excluding dietary misreporters (online supplementary material, Appendices E and F).

## Discussion

### Summary of main findings

This nationally representative study of British schoolchildren found that school meals are of a higher nutritional quality than packed lunches, but neither have reached optimal nutritional composition. Children consuming school meals were more likely to meet food and nutrient recommendations compared with those taking packed lunches, especially in the provision of vegetables and limiting the consumption of sweet and savoury snacks. However, more than 30 % of school meals still contained a sweet snack. The quality of school meals was also found to decline with age while packed lunches remained of similar, relatively poor nutritional quality across age groups. Compared with school meals consumed by younger children, those consumed by older children were less likely to contain recommended amounts of fruits and vegetables, and an increasing proportion contained sweet and savoury snacks compared with younger children.

### Strengths and limitations

Strengths of this study include being the first, large and nationally representative, UK-wide analysis providing a comprehensive assessment of the diet and nutritional quality of school meals and packed lunches, including academic KS of schoolchildren. In addition, the NDNS uses a high-quality, validated 4-d food diary accounting for within-person day-to-day consumption variability, although a maximum of 3 d/child were used in this assessment due to the inclusion of a weekend day^(2)^. The dietary assessment used is also highly detailed, allowing for a detailed description of the food and nutrient content of the lunches.

There are several limitations which must be considered. First, the food diaries of children less than 11 years were recorded by a guardian, whereas students aged 12 and over completed their own food diary which may have resulted in systematic differences contributing to the differences seen between primary and secondary school students. As self-reported energy intake is typically under-reported^(33)^, our findings might under-estimate dietary intakes. In sensitivity analyses, we excluded 485 participants who were estimated to misreport their diet relative to their estimated energy requirements and found that this limitation did not bias the results. Second, 75 % of participants recorded which meal type they consumed on each school day; however, 25 % did not have these data available or recorded more than one meal type over the study period. Where meal type data were unavailable, the survey question on usual meal type consumption (described above) was used to estimate meal type. Where more than one meal type was recorded (5 %), the most frequent meal type was used. There was high similarity between the two methods of meal type definition. We performed sensitivity analyses to ensure that differences in meal-type definition did not impact the conclusions from the analysis. Third, students who consumed lunch outside the school premises (e.g. at a shop or café) were excluded from the analysis as it could not be confirmed if this was part of a school day or a holiday. The approach used ensures only term-time intakes are included. Fourth, the number of days recorded between participants varied, with 15 % of the sample (*n* 457) only recording one lunch, which may mean their results were less representative of their total diet and there was more measurement error where fewer days were recorded. There were also some policy changes over the study period, including the introduction of food-based school standards in 2015 and the UIFSM. We accounted for variation by adjusting for survey year. Moreover, the UIFSM scheme only affected KS-1 students, as we observed that KS-2 children also had preferable dietary intakes, the differences do not appear to be driven by the UIFSM programme. Finally, low annual sample sizes meant that the survey years were pooled to increase sample size and allow a detailed nutritional characterisation of meal types by academic KS. We were therefore not able to stratify by age and analyse trends overtime.

### Relationship to prior knowledge

Our study is unique in comparing school lunches across a wide range of age groups, showing that the nutritional quality of lunches in younger children was impacted by meal type, while, in older children, the smaller effects of meal type on nutrition might be due to the declining quality of school meals at higher KS. These conclusions are congruent with previous literature reporting a consistent benefit of school meals in primary schools^(9,18–20)^, with similar but smaller effect sizes seen in secondary schools^(22–25)^. Furthermore, although school food provision varies substantially between countries^(34)^, school meals have also been associated with improved dietary quality in other countries such as the USA^(35)^ and Denmark^(36)^. However, only a few studies have disaggregated this association by KS, which broadly reflect age groups. For instance, a study conducted before 2009 found differences in the nutrient intake of school meals and packed lunches in KS-1 children compared with KS-2; however, the analysis did not include children over 12 years old^(18)^. For example, salt intake was lower for both KS-1 and KS-2 children taking a school meal, but fat intake was only lower for KS-1 children. Compared with this analysis, our study found more consistent benefits of taking a school meal for both KS-1 and KS-2 children, with lower fat, sugar and salt content of school meals.

### Interpretation and implications

There are multiple mechanisms which might explain why the nutritional gap between school meals and packed lunches may reduce for secondary schoolchildren. First, there is evidence that the School Food Standards are not applied in many secondary schools. Research has shown that the School Food Standards have improved children’s eating^(15,25)^; however, the standards do not legally apply to academies in England (schools which are not under local government control) formed between 2010 and 2014, estimated to be up to 50 % of all secondary schools^(37)^. Additionally, while the standards are compulsory, they are not formally monitored in England. As such, 60 % of secondary schools may not comply with the School Food Standards, partly driven by reduced funding and cost-saving measures^(38)^. However, in Northern Ireland, between December 2006 and March 2011, the Education and Training Inspectorate evaluated the progress made in the implementation of the School Food Standards by visiting 394 schools, including secondary schools, where they found that good compliance was present with the school food standards^(10,39)^. Greater formal monitoring should be in place, specifically in secondary school to encourage the implementation of the School Food Standards. Second, secondary schoolchildren have increased choice and autonomy over their food consumption at school; therefore, individual choice may play a larger role in their diet. Qualitative research has highlighted that compared with younger children, the increased independence adolescents experience results in their food choices being increasingly influenced by preference, convenience and social factors over nutritional quality^(40–42)^. Consequently, despite some School Food Standard compliant options being on offer, the majority of secondary schoolchildren still choose the least healthy lunch options^(40,43)^. While the choice of meal type might be associated with socio-economic factors, we did not see differences in socio-economic characteristics by meal type across KS.

The finding that school meals were of a higher nutritional quality than packed lunches, but neither reached optimal nutritional composition, indicates that steps should be taken to improve student nutrition. In our study, packed lunches were shown to be nutritionally inferior to school meals among students in KS-1 and 2, demonstrating the School Food Standards to be effective at this school phase. Constraints on parents’ time combined with influence from the child^(44)^ increase the likelihood that families choose child-targeted convenience foods for packed lunches^(21)^. These foods are more likely to be of worse nutritional quality than adult versions^(45)^ or less processed foods^(46)^. Recently, there have been renewed calls to reform the School Food Standards, as they are not optimal^(47)^. Scotland, for example, has addressed these calls by revising and publishing updated School Food Standards for primary and secondary schoolchildren which focused on implementing evidence-based changes to support the reduction of obesity, reduced health inequalities and dental health^(48)^. Key changes include tailored criteria for primary *v*. secondary schoolchildren, a reform to the definition of ‘sugar’ with several key changes aimed at reducing overall free sugars in schoolchildren’s meals, promotion of full portion of fruit and vegetables rather than diversity, increased fibre, reduced red and processed meats and daily energy guidelines^(48)^. However, approximately 50 % of students are taking packed lunches that are not addressed by compulsory nutritional standards and while the changes introduced are positive, they focus on key areas of concern identified for those taking packed lunches such as higher levels of sugar and fat and lower consumption of vegetables. Our results highlight the importance of the continued and increased promotion of the uptake of school meals to improve the nutritional quality of children’s diet and reduce associated inequalities. Furthermore, these findings draw attention to the dietary inequalities which may be present outside of term time and raise concern over the issue of holiday hunger^(49)^. The School Food Plan^(50)^ proposes the adoption of a ‘whole-school approach’ which encompasses the provision of nutritious foods while also creating an environment that promotes nutritionally optimal decisions for all lunches. While there are simple changes that can be made by schools, more concerted efforts are needed to enable schools to make large-scale, permanent improvements to their school food environment.

## Conclusion

Between 2008 and 2017, school meals consumed by British children were found to be nutritionally superior to packed lunches. However, school meals were not yet optimal and declined in quality with age. These findings highlight the necessity for robust public health measures to improve student nutrition with a particular focus on secondary schoolchildren.

## Supplementary material

For supplementary material accompanying this paper visit https://doi.org/10.1017/S1368980022001355. click here to view supplementary material
