# Supplementary material for: Dietary quality of school meals and packed lunches: a national study of primary and secondary schoolchildren in the UK
Source: Public Health Nutr. 2022 Jun 1;26(2):425–36. doi: 10.1017/S1368980022001355 (PMC13076075; doi:10.1017/S1368980022001355)
Supplement: Supplementary file 1 [file S1368980022001355sup.zip › S1368980022001355sup001.docx]

STROBE Statement—Checklist of items that should be included in reports of ***cross-sectional studies***

|  | Item No | Recommendation | Page No |
| --- | --- | --- | --- |
| **Title and abstract** | 1 | 1. Indicate the study’s design with a commonly used term in the title or the abstract 2. State the Dietary/Nutritional Assessment Method(s) Used in the Title, Abstract, or Keywords | 1 |
|  |  | (*c*) Provide in the abstract an informative and balanced summary of what was done and what was found | 1 |
| Introduction | | | |
| Background/rationale | 2 | Explain the scientific background and rationale for the investigation being reported | 2-3 |
| Objectives | 3 | State specific objectives, including any prespecified hypotheses | 3 |
| Methods | | | |
| Study design | 4 | Present key elements of study design early in the paper | 3 |
| Setting | 5 | Describe the setting, locations, and relevant dates, including periods of recruitment, exposure, follow-up, and data collection  Describe Any Characteristics of Study Settings That Might Affect the Dietary Intake or Nutritional Status of the Participants, If Applicable | 3 |
| Participants | 6 | 1. Give the eligibility criteria, and the sources and methods of selection of participants 2. Report Particular Dietary, Physiological, or Nutritional Characteristics That Were Considered When Selecting the Participants | 3 |
| Variables | 7 | 1. Clearly define all outcomes, exposures, predictors, potential confounders, and effect modifiers. Give diagnostic criteria, if applicable 2. Clearly Define Foods, Food Groups, Nutrients, or Other Food Components | 3-4 |
| Data sources/ measurement | 8* | 1. For each variable of interest, give sources of data and details of methods of assessment (measurement). Describe comparability of assessment methods if there is more than one group 2. Describe the Dietary Assessment Method(s) 3. Describe and Justify Food Composition Data Used 4. Report on the Validity of the Dietary or Nutritional Assessment Methods and Any Internal or External Validation Used in the Study, If Applicable | *3-4* |
| Bias | 9 | 1. Describe any efforts to address potential sources of bias 2. Report How Bias in Dietary or Nutritional Assessment, E.g., Misreporting, Changes in Habits as a Result of Being Measured, and Data Imputation from Other Sources, Was Addressed | 4-5 |
| Study size | 10 | Explain how the study size was arrived at | 3 |
| Quantitative variables | 11 | Explain how quantitative variables were handled in the analyses. If applicable, describe which groupings were chosen and why  Explain the Categorization of Dietary/Nutritional Data (E.g., Use of N-tiles and Handling of Nonconsumers) and the Choice of Reference Category, If Applicable | 4-5 |
| Statistical methods | 12 | (*a*) Describe all statistical methods, including those used to control for confounding | 4-5 |
|  |  | (*b*) Describe any methods used to examine subgroups and interactions | 4-5 |
|  |  | (*c*) Explain how missing data were addressed | 4 |
|  |  | (*d*) If applicable, describe analytical methods taking account of sampling strategy | 5 |
|  |  | (*e*) Describe any sensitivity analyses  (*f*) Describe and Justify the Method for Energy Adjustments, Intake Modeling and Use of Weighting Factors, If Applicable | 5 |
| Results | | | |
| Participants | 13* | (a) Report numbers of individuals at each stage of study—eg numbers potentially eligible, examined for eligibility, confirmed eligible, included in the study, completing follow-up, and analysed | 5 |
|  |  | (b) Give reasons for non-participation at each stage | 3 |
|  |  | (c) Consider use of a flow diagram |  |
|  |  | Report the Number of Individuals Excluded Based on Missing, Incomplete, or Implausible Dietary/Nutritional Data | 3-4 |
| Descriptive data | 14* | (a) Give characteristics of study participants (eg demographic, clinical, social) and information on exposures and potential confounders | 5 |
|  |  | (b) Indicate number of participants with missing data for each variable of interest | n/a |
|  |  | Give the Distribution of Participant Characteristics across the Exposure Variables If Applicable. Specify If Food Consumption of Total Population or Consumers Only Were Used to Obtain Results | 5 |
| Outcome data | 15* | Report numbers of outcome events or summary measures | 5-6 |
| Main results | 16 | (*a*) Give unadjusted estimates and, if applicable, confounder-adjusted estimates and their precision (eg, 95% confidence interval). Make clear which confounders were adjusted for and why they were included | 4 |
|  |  | (*b*) Report category boundaries when continuous variables were categorized | 4 |
|  |  | (*c*) If relevant, consider translating estimates of relative risk into absolute risk for a meaningful time period | n/a |
|  |  | Specify If Nutrient Intakes Are Reported with or without Inclusion of Dietary Supplement Intake, If Applicable | 3 |
| Other analyses | 17 | Report other analyses done—eg analyses of subgroups and interactions, and sensitivity analyses | 6 |
|  |  | Report Any Sensitivity Analysis (E.g., Exclusion of Misreporters or Outliers) and Data Imputation, If Applicable | 6-7 |
| Discussion | | | |
| Key results | 18 | Summarise key results with reference to study objectives | 7 |
| Limitations | 19 | Discuss limitations of the study, taking into account sources of potential bias or imprecision. Discuss both direction and magnitude of any potential bias | 7-8 |
|  |  |  |  |
| Interpretation | 20 | Give a cautious overall interpretation of results considering objectives, limitations, multiplicity of analyses, results from similar studies, and other relevant evidence | 8 |
|  |  | Report the Nutritional Relevance of the Findings, Given the Complexity of Diet or Nutrition as an Exposure | 8-9 |
| Generalisability | 21 | Discuss the generalisability (external validity) of the study results | 7 |
| Other information | | | |
| Funding | 22 | Give the source of funding and the role of the funders for the present study and, if applicable, for the original study on which the present article is based | Title page |
|  |  | Describe the Procedure for Consent and Study Approval from Ethics Committee(s) | Title page |
|  |  | Provide Data Collection Tools and Data as Online Material or Explain How They Can Be Accessed | Title page |

*Give information separately for exposed and unexposed groups.

**Note:** An Explanation and Elaboration article discusses each checklist item and gives methodological background and published examples of transparent reporting. The STROBE checklist is best used in conjunction with this article (freely available on the Web sites of PLoS Medicine at http://www.plosmedicine.org/, Annals of Internal Medicine at http://www.annals.org/, and Epidemiology at http://www.epidem.com/). Information on the STROBE Initiative is available at www.strobe-statement.org.
