# Supplementary material for: Dietary quality of school meals and packed lunches: a national study of primary and secondary schoolchildren in the UK
Source: Public Health Nutr. 2022 Jun 1;26(2):425–36. doi: 10.1017/S1368980022001355 (PMC13076075; doi:10.1017/S1368980022001355)
Supplement: Supplementary file 1 [file S1368980022001355sup.zip › S1368980022001355sup002.pdf]

Appendix A. Operational definitions of food and nutrient outcome variables[42].

Outcome	Units	Operational Definition
**Food Groups**		
Fruit	g	Fruit, including from composite dishes. Includes canned but does not include dried fruit
Vegetables	g	Any vegetable, including from composite dishes
Protein-rich foods	g	Any meat, fish, egg, or beans, including from composite dishes
Wholemeal products	g	Any wholemeal starch product (e.g. bread, pasta, rice)
Dairy (milk, yoghurt, cheese)	g	Any dairy milk, yogurt, or cheese including from composite dishes.
Savoury snacks	g	Any crisps or savoury snacks (Includes all potato and cereal based snacks, popcorn (not sweet), twiglets, pretzels, pork scratchings)
Sweet snacks	g	Any manufactured/retail or homemade biscuit, any sugar or chocolate confectionery, buns, cakes, pastries, fruit pies, cereal based milk puddings, sponge puddings, and other cereal based puddings (manufactured/homemade)
**Nutrients**		
Fibre	g	AOAC method of measuring fibre
Vitamin C	mg	Any vitamin C
Calcium	mg	Any calcium
Iron	mg	Sum of all haem iron and non haem iron
Non-milk extrinsic sugar	g	Sum of all sugars in fruit juices, table sugar, honey, sucrose and glucose syrups added to foods
Fat	g	Sum of all fatty acids
Saturated fatty acids	g	Any saturated fatty acids
Salt	g	Any salt

Appendix B. Nutrient Recommendations for primary and secondary students.

Nutrient type (units)	Recommendation	
	Primary	Secondary
**Nutrients with minimum recommendation***		
Fibre (g)	4.2	5.2
Vitamin C (mg)	10.5	14.0
Calcium (mg)	193.0	350.0
Iron (mg)	3.0	5.2
**Nutrients with maximum recommendation†**		
NMES (g)	15.5	18.9
SFA (g)	6.5	7.9
Salt (g)	1.2	1.8

*Minimum recommendation refers to nutrients that must be at a minimum intake to meet the 2008/09 English nutrient-based standards[26].

†Maximum recommendation refers to nutrients that must remain below a certain level to meet the 2008/09 English nutrient-based standards[26].

*Minimum recommendation refers to nutrients that must be at a minimum intake to meet the 2008/09 English nutrient-based standards[26].

†Maximum recommendation refers to nutrients that must remain below a certain level to meet the 2008/09 English nutrient-based standards[26].

Appendix C. Sensitivity analyses testing different specification of meal type definitions **for** the likelihood of packed lunches vs. school meals in containing each food group, by academic key stage.

	**Model 1**		**Model 2**		**Model 3**	
	**OR**	**[95% CI]**	**OR**	**[95% CI]**	**OR**	**[95% CI]**
**Fruit**						
Key stage 1	2.9***	[1.9,4.5]	2.9***	[1.9,4.6]	1.8***	[1.4,2.4]
Key stage 2	2.7***	[1.9,3.8]	2.8***	[1.9,4.0]	1.8***	[1.4,2.2]
Key stage 3	4.0***	[2.5,6.1]	4.0***	[2.6,6.3]	2.5***	[1.7,3.8]
Key stage 4	2.1*	[1.2,3.7]	2.7**	[1.5,5.0]	1.9*	[1.1,3.1]
**Vegetables**						
Key stage 1	0.0***	[0.0,0.1]	0.1***	[0.0,0.1]	0.1***	[0.1,0.2]
Key stage 2	0.1***	[0.1,0.1]	0.1***	[0.1,0.1]	0.2***	[0.2,0.3]
Key stage 3	0.2***	[0.1,0.4]	0.2***	[0.1,0.3]	0.4***	[0.3,0.6]
Key stage 4	0.2***	[0.1,0.4]	0.3***	[0.1,0.5]	0.3***	[0.2,0.6]
**Protein-rich foods**						
Key stage 1	0.3***	[0.2,0.5]	0.2***	[0.1,0.4]	0.5***	[0.4,0.7]
Key stage 2	0.4***	[0.2,0.6]	0.3***	[0.2,0.6]	0.4***	[0.3,0.6]
Key stage 3	0.6	[0.4,1.0]	0.7	[0.4,1.0]	1	[0.7,1.6]
Key stage 4	0.6	[0.3,1.2]	0.8	[0.4,1.5]	0.6	[0.3,1.0]
**Wholemeal products**						
Key stage 1	7.4***	[4.7,11.7]	7.3***	[4.8,11.4]	3.2***	[2.3,4.3]
Key stage 2	6.8***	[4.4,10.5]	4.6***	[3.1,6.9]	4.5***	[3.2,6.4]
Key stage 3	3.3***	[2.0,5.5]	2.9***	[1.7,4.9]	2.7***	[1.7,4.3]
Key stage 4	2.4*	[1.1,4.9]	2.1	[1.0,4.3]	2.5**	[1.3,4.8]
**Dairy**						
Key stage 1	2.1***	[1.4,3.1]	1.9**	[1.3,2.7]	1.5**	[1.2,1.9]
Key stage 2	1.6*	[1.1,2.2]	1.4	[1.0,2.0]	1.2	[1.0,1.5]
Key stage 3	0.9	[0.6,1.4]	0.9	[0.6,1.4]	1	[0.7,1.5]
Key stage 4	0.9	[0.5,1.6]	0.8	[0.4,1.5]	0.6	[0.3,1.0]
**Savoury snacks**						
Key stage 1	22.6***	[11.5,44.3]	16.5***	[9.4,29.0]	9.8***	[4.5,21.2]
Key stage 2	11.7***	[7.1,19.4]	10.7***	[6.4,17.9]	8.2***	[4.6,14.7]
Key stage 3	9.0***	[5.1,15.7]	8.7***	[4.9,15.3]	7.9***	[3.9,15.9]
Key stage 4	7.2***	[3.1,16.4]	7.3***	[3.1,17.3]	6.4*	[1.3,32.4]
**Sweet snacks**						
Key stage 1	5.2***	[2.4,11.1]	5.7***	[2.6,12.2]	2.5**	[1.3,4.8]
Key stage 2	8.1***	[4.7,13.8]	8.0***	[4.6,13.8]	6.2***	[3.6,10.4]
Key stage 3	2.2**	[1.2,3.8]	2.2**	[1.2,3.9]	2.2**	[1.4,3.5]
Key stage 4	3.2**	[1.5,6.8]	2.5*	[1.1,6.0]	3.5**	[1.5,7.9]

*P<0.05 **P<0.01 **P<0.001; OR – Odds ratio; CI – confidence interval

Model 1 (main analysis) – Meal type was defined as the most frequent location in the food diary, if meal type could not be determined from the food diary, school lunch preference was used. Total sample *n*=3001

Model 2 (sensitivity analysis) – Meal type was determined from the food diary, if participants recorded more than one location or their meal type could not be determined from the food diary school lunch preference was used. Total sample *n*= 2,986, *n*=15 removed

Model 3 (sensitivity analysis) – Meal type definition was taken from the food diary, if participants recorded more than one location or their meal type could not be determined from the food diary they were excluded. Total sample *n*=1,671

Appendix D. Sensitivity analyses testing different specification of meal type definitions for the likelihood of packed lunches vs. school meals in meeting nutrient recommendations, by academic key stage.

	Model 1		Model 2		Model 3	
	**OR**	**[95% CI]**	**OR**	**[95% CI]**	**OR**	**[95% CI]**
**Fibre**						
Key stage 1	0.6**	[0.4,0.8]	0.5***	[0.4,0.8]	0.6***	[0.5,0.8]
Key stage 2	0.5***	[0.3,0.6]	0.5***	[0.3,0.7]	0.6***	[0.5,0.8]
Key stage 3	0.9	[0.6,1.4]	0.9	[0.5,1.4]	1.2	[0.8,1.7]
Key stage 4	1	[0.5,1.8]	0.9	[0.5,1.7]	0.9	[0.5,1.6]
**Vitamin C**						
Key stage 1	1	[0.7,1.5]	1.1	[0.8,1.6]	1.1	[0.8,1.4]
Key stage 2	0.9	[0.7,1.3]	0.9	[0.6,1.2]	0.9	[0.7,1.1]
Key stage 3	1.2	[0.8,1.8]	1.2	[0.8,1.7]	1	[0.7,1.4]
Key stage 4	0.8	[0.4,1.4]	0.6	[0.3,1.0]	0.9	[0.5,1.5]
**Calcium**						
Key stage 1	2.4***	[1.7,3.4]	2.7***	[1.9,3.9]	1.8***	[1.4,2.3]
Key stage 2	3.0***	[2.1,4.2]	2.6***	[1.9,3.7]	1.9***	[1.5,2.4]
Key stage 3	1.1	[0.6,2.0]	1.1	[0.6,1.9]	1.1	[0.6,1.9]
Key stage 4	0.8	[0.4,1.9]	0.6	[0.3,1.4]	0.6	[0.3,1.2]
**Iron**						
Key stage 1	0.9	[0.5,1.6]	0.9	[0.5,1.6]	1	[0.7,1.4]
Key stage 2	1.4	[0.8,2.2]	1.2	[0.7,1.9]	1.3	[1.0,1.7]
Key stage 3	2.9	[0.9,9.9]	3.9	[0.8,18.7]	9.2**	[2.1,41.4]
Key stage 4	7.9	[0.5,135.8]	1	[1.0,1.0]		
**NMES**						
Key stage 1	0.4***	[0.3,0.6]	0.3***	[0.2,0.5]	0.6***	[0.4,0.7]
Key stage 2	0.3***	[0.2,0.5]	0.4***	[0.3,0.6]	0.6***	[0.5,0.7]
Key stage 3	1.3	[0.9,2.0]	1.3	[0.9,2.0]	1.1	[0.8,1.5]
Key stage 4	1	[0.5,1.8]	1.3	[0.7,2.4]	1.1	[0.6,1.9]
**Saturated Fat**						
Key stage 1	0.6**	[0.4,0.9]	0.7*	[0.5,1.0]	0.8*	[0.6,1.0]
Key stage 2	0.5***	[0.3,0.6]	0.6***	[0.4,0.8]	0.6***	[0.5,0.7]
Key stage 3	1.5	[1.0,2.3]	1.5	[0.9,2.3]	1.1	[0.8,1.6]
Key stage 4	1	[0.5,2.0]	1.3	[0.7,2.7]	1.7	[0.9,3.1]
**Sodium**						
Key stage 1	0.3***	[0.2,0.4]	0.3***	[0.2,0.4]	0.5***	[0.4,0.7]
Key stage 2	0.2***	[0.1,0.3]	0.3***	[0.2,0.4]	0.4***	[0.3,0.5]
Key stage 3	0.7	[0.5,1.1]	0.6*	[0.4,1.0]	0.7*	[0.5,1.0]
Key stage 4	0.6	[0.3,1.1]	0.6	[0.3,1.3]	1.1	[0.7,2.0]

*P<0.05 **P<0.01 **P<0.001; OR – Odds ratio; CI – confidence interval

Model 1 (main analysis) – Meal type was defined as the most frequent location in the food diary, if meal type could not be determined from the food diary, school lunch preference was used. Total sample *n*=3001

Model 2 (sensitivity analysis) – Meal type was determined from the food diary, if participants recorded more than one location or their meal type could not be determined from the food diary school lunch preference was used. Total sample *n*= 2,986, *n*=15 removed

Model 3 (sensitivity analysis) – Meal type definition was taken from the food diary, if participants recorded more than one location or their meal type could not be determined from the food diary they were excluded. Total sample *n*=1,671

Appendix E. Sensitivity analyses testing the impact of energy mis-reporting on the likelihood of packed lunches vs. school meals in containing each food group, by academic key stage.

	Model 1		Model 2	
	**OR**	**[95% CI]**	**OR**	**[95% CI]**
**Fruit**				
Key stage 1	2.9***	[1.9,4.5]	3.0***	[1.9,4.7]
Key stage 2	2.7***	[1.9,3.8]	2.9***	[2.0,4.3]
Key stage 3	4.0***	[2.5,6.1]	4.3***	[2.6,7.0]
Key stage 4	2.1*	[1.2,3.7]	1.8	[0.9,3.7]
**Vegetables**				
Key stage 1	0.0***	[0.0,0.1]	0.0***	[0.0,0.1]
Key stage 2	0.1***	[0.1,0.1]	0.1***	[0.1,0.1]
Key stage 3	0.2***	[0.1,0.4]	0.2***	[0.1,0.4]
Key stage 4	0.2***	[0.1,0.4]	0.2***	[0.1,0.5]
**Protein-rich foods**				
Key stage 1	0.3***	[0.2,0.5]	0.3***	[0.2,0.5]
Key stage 2	0.4***	[0.2,0.6]	0.4***	[0.2,0.7]
Key stage 3	0.6	[0.4,1.0]	0.6	[0.4,1.1]
Key stage 4	0.6	[0.3,1.2]	0.6	[0.3,1.5]
**Wholemeal products**				
Key stage 1	7.4***	[4.7,11.7]	6.9***	[4.4,11.0]
Key stage 2	6.8***	[4.4,10.5]	6.5***	[4.1,10.3]
Key stage 3	3.3***	[2.0,5.5]	4.2***	[2.2,7.8]
Key stage 4	2.4*	[1.1,4.9]	2	[0.8,4.8]
**Dairy**				
Key stage 1	2.1***	[1.4,3.1]	2.0***	[1.3,2.9]
Key stage 2	1.6*	[1.1,2.2]	1.7**	[1.1,2.4]
Key stage 3	0.9	[0.6,1.4]	1	[0.6,1.6]
Key stage 4	0.9	[0.5,1.6]	0.8	[0.4,1.7]
**Savoury snacks**				
Key stage 1	22.6***	[11.5,44.3]	23.1***	[11.3,47.5]
Key stage 2	11.7***	[7.1,19.4]	12.5***	[7.3,21.6]
Key stage 3	9.0***	[5.1,15.7]	9.4***	[5.1,17.6]
Key stage 4	7.2***	[3.1,16.4]	8.2***	[2.9,22.7]
**Sweet snacks**				
Key stage 1	5.2***	[2.4,11.1]	5.3***	[2.4,11.5]
Key stage 2	8.1***	[4.7,13.8]	9.2***	[5.2,16.3]
Key stage 3	2.2**	[1.2,3.8]	2.3**	[1.2,4.4]
Key stage 4	3.2**	[1.5,6.8]	3.7**	[1.4,9.6]

*P<0.05 **P<0.01 **P<0.001; OR – Odds ratio; CI – confidence interval

Model 1 (main analysis) – Fully adjusted will all participants included *n*=3001
Model 2 (sensitivity analysis) – Fully adjusted with all energy mis-reporters excluded (*n*=445), leaving a sample of *n*=2,556

Appendix F. Sensitivity analyses testing the impact of energy mis-reporting on the likelihood of packed lunches vs. school meals in meeting nutrient recommendations, by academic key stage.

	Model 1		Model 2	
	**OR**	**[95% CI]**	**OR**	**[95% CI]**
**Fibre**				
Key stage 1	0.6**	[0.4,0.8]	0.6**	[0.4,0.8]
Key stage 2	0.5***	[0.3,0.6]	0.5***	[0.3,0.6]
Key stage 3	0.9	[0.6,1.4]	1	[0.6,1.7]
Key stage 4	1	[0.5,1.8]	0.8	[0.4,1.6]
**Vitamin C**				
Key stage 1	1	[0.7,1.5]	1	[0.7,1.5]
Key stage 2	0.9	[0.7,1.3]	0.9	[0.7,1.3]
Key stage 3	1.2	[0.8,1.8]	1.1	[0.7,1.8]
Key stage 4	0.8	[0.4,1.4]	0.8	[0.4,1.7]
**Calcium**				
Key stage 1	2.4***	[1.7,3.4]	2.4***	[1.7,3.4]
Key stage 2	3.0***	[2.1,4.2]	3.0***	[2.1,4.3]
Key stage 3	1.1	[0.6,2.0]	1.3	[0.7,2.6]
Key stage 4	0.8	[0.4,1.9]	0.7	[0.3,1.7]
**Iron**				
Key stage 1	0.9	[0.5,1.6]	0.9	[0.5,1.6]
Key stage 2	1.4	[0.8,2.2]	1.3	[0.8,2.1]
Key stage 3	2.9	[0.9,9.9]	5.1*	[1.0,25.2]
Key stage 4	7.9	[0.5,135.8]	1	[1.0,1.0]
**NMES**				
Key stage 1	0.4***	[0.3,0.6]	0.4***	[0.3,0.6]
Key stage 2	0.3***	[0.2,0.5]	0.3***	[0.2,0.5]
Key stage 3	1.3	[0.9,2.0]	1.4	[0.9,2.2]
Key stage 4	1	[0.5,1.8]	0.9	[0.4,1.9]
**Saturated Fat**				
Key stage 1	0.6**	[0.4,0.9]	0.6**	[0.4,0.9]
Key stage 2	0.5***	[0.3,0.6]	0.5***	[0.3,0.7]
Key stage 3	1.5	[1.0,2.3]	1.2	[0.8,2.0]
Key stage 4	1	[0.5,2.0]	1	[0.4,2.2]
**Sodium**				
Key stage 1	0.3***	[0.2,0.4]	0.3***	[0.2,0.4]
Key stage 2	0.2***	[0.1,0.3]	0.2***	[0.1,0.3]
Key stage 3	0.7	[0.5,1.1]	0.6*	[0.4,1.0]
Key stage 4	0.6	[0.3,1.1]	0.5	[0.2,1.1]

*P<0.05 **P<0.01 **P<0.001; OR – Odds ratio; CI – confidence interval

Model 1 (main analysis) – Fully adjusted will all participants included *n*=3001
Model 2 (sensitivity analysis) – Fully adjusted with all energy mis-reporters excluded (*n*=445), leaving a sample of *n*=2,556
